# The Early Home Treatment of Consumption

**Published:** 1901-12-14

**Authors:** 


### THE EARLY HOME TREATMENT OF CONSUMPTION.

In a discussion which recently took place at the New York Post-Graduate Clinical Society on the home treatment of consumption, the most important point raised was in regard to the necessity of dealing seriously with the cases as soon as the nature of the disease is discovered. Dr. Leonard Weber said that it was surprising that cases of recent tuberculosis coming on with more or less febrile disturbance of the system were so often not sent to bed. Such cases should, he thought, be kept in bed for some weeks, in fact until the acute stage was passed. Unfortunately these patients do not feel very ill at the onset of tuberculous invasion, and even before the first visit to the doctor quite a considerable area of lung may be involved. If such a patient felt as ill as one attacked with a quinsey, more cases would be recognised in early stages. The first rule then that he lays down is that in a case of fresh tuberculous infection the patient should go to bed and stay there until a practically normal temperature is attained. "Rest-cure at the outset, to be repeated at intervals according to the circumstances of the case, and careful nursing are essential for successful treatment." At the present day there seems much reason for emphasising this advice. On every hand the advantage, and even the necessity, of sanatorium treatment is being urged, and much that is being said in its favour is absolutely true. If on the discovery that a patient is affected with early tuberculosis one could by the waving of a wand transport him to some well organised sanatorium, one would do him an infinite benefit. Unfortunately this cannot be, and it is to be feared that the dependence which is coming to be placed upon sanatorium treatment leads not infrequently to the patient not receiving all the care which ought to be his while arrangements are being made for his reception at some institution. When the order has been given and the sentence has been once pronounced that the patient is to go away, both he and his friends, perhaps not unnaturally, regard all else but sanatorium treatment as mere makeshift. There is an interregnum between the self rule which is to be given up and the institution rule which is so soon to be submitted to, and this interregnum is a dangerous period. Moreover, it is often far more prolonged than is anticipated. The half dozen sanatoria which can be really recommended are probably full, while in regard to the scores of others endless inquiries have to be made and family discussions held. Meanwhile the weeks slide by, and the patient, just at the period of his disease when he ought to be under the most strict discipline and observation, kicks against all treatment and restriction. Will not his "real" treatment begin in another week's time, and what is the good of bothering to get into new ways when all will have to be upset again so soon? That is the way the patient argues; while sometimes, if he is a fool—and there are still a good many fools in this world—he may actually go so far as to give way to injudicious self-indulgence before being "shut up" as he calls it. Nor is it always easy for the family doctor to shake himself free from the enervating uncertainty of the interregnum. He cannot feel quite sure where his patient is going, he cannot know how far the "real treatment" when it is begun will tally with what he knows to be best at the present moment, and in the midst of all this uncertainty it is by no means easy to speak firmly and persuade people to upset their houses and make fresh arrangements as to nursing, feeding, and the rest, "just for a few days," as they say and as they hope. Many a case slips beyond the boundary line during that wretched time while the patient is waiting for his turn at the sanatorium, when, although he is ailing he is not ill, and when to all concerned it seems hardly worth while to begin a definite treatment. We therefore fully endorse what Dr. Leonard Weber insists upon in regard to the necessity of serious treatment during the early period of commencing tuberculosis. The patient must rest mostly in bed, his room must be well above ground, must admit plenty of air and light, and be easy to ventilate. It must contain but little furniture and must be capable of being easily cleaned down with a damp cloth. The heating should be by an open fire, proper enamelled or glass spittoons, or those made of paper must be used for the reception of the expectoration. The paper ones must, of course, be burned when used, while the others must be systematically disinfected either by strong antiseptics or by boiling. The windows must be kept open so that the air of the room shall be as pure as that outside, and all hygienic rules must be attended to. Food must be given, if necessary, in small quantities every two or three hours, but as soon as possible in the shape of meals four times a day. During the acute febrile stage, if such should exist, the patient must be sponged with alcohol and water three times daily, and his linen be changed as often as required, while when he has improved and is convalescent he should take a cool sponge bath every morning, standing in a rubber tub before the washstand on which is placed a large basin full of cool water; the time occupied by the sponging and drying being from two to five minutes. Afterwards, when the "rest-cure" part of the business is over, he takes a douche or rain bath (warm or tepid followed by cool or cold) every morning. What is important then is that when tuberculosis is discovered, even when it is intended to send the patient to a sanatorium, no time should be lost in putting him into the best position for recovery. If the treatment is to be undertaken at home, and in many homes this is quite possible if the doctor be confident and the patient submissive, it should be commenced seriously from the beginning; while if the sanatorium treatment is aimed at, the serious treatment of the patient should still be commenced at home pending the completion of arrangements, for these arrangements often take some time, and if there is any pyrexia going on this period of interregnum is full of danger to the patient.

